# Gastric and small bowel metastases from lung adenocarcinoma: A case report

**DOI:** 10.1097/MD.0000000000040058

**Published:** 2024-10-11

**Authors:** Yan Wang, Li Xu, Jun-Lei Wu, Chao-Qun Wang

**Affiliations:** a Department of Medical Oncology, Affiliated Dongyang Hospital of Wenzhou Medical University, Dongyang, Zhejiang, China; b Interventional Radiology Department, Affiliated Dongyang Hospital of Wenzhou Medical University, Dongyang, Zhejiang, China; c Department of Pathology, Affiliated Dongyang Hospital of Wenzhou Medical University, Dongyang, Zhejiang, China.

**Keywords:** case report, gastric, lung cancer, metastasis, small bowel

## Abstract

**Rationale:**

Metastatic tumors in the stomach and small bowel are rare. This article reports a case of lung adenocarcinoma metastasizing to the stomach and small bowel.

**Patient concerns:**

This case study presents the medical history of a 41-year-old male construction worker with a 1-month-long cough and slight chest discomfort. Imaging revealed a tumor in the right middle lobe of the lung, with metastasis to lymph nodes in the mediastinum and right hilar region, as well as a mass in the stomach’s greater curvature and multiple lymph node metastases in the abdomen. Thirty-five days after the initial consultation, the patient exhibited worsening symptoms of vomiting and melena. A follow-up computed tomography scan revealed small bowel metastasis, leading to secondary intestinal obstruction and intussusception.

**Diagnoses:**

Biopsies confirmed poorly differentiated adenocarcinoma in the lung and stomach, with immunohistochemistry supporting a diagnosis of lung adenocarcinoma. Genetic testing showed no mutations or amplification in various genes.

**Interventions:**

The patient received interventional hemostatic treatment; however, the efficacy of the intervention was poor.

**Outcomes:**

The patient experienced worsening gastrointestinal symptoms. Despite attempted intervention, the patient ultimately died 78 days after seeking medical attention.

**Lessons:**

The case of lung cancer metastasizing to the stomach and small bowel presented in this article demonstrates high invasiveness and rapid progression. Combined with literature reports, this type of metastasis often indicates a poor prognosis for patients. The long-term benefits of surgical resection remain unclear, and further analysis will be needed with more cases and data in the future.

## 1. Introduction

Metastatic tumors occurring in the stomach and small bowel are considered uncommon occurrences in clinical practice.^[1–4]^ This article focuses on a unique case of lung adenocarcinoma that metastasized to both the stomach and small bowel, presenting a rare and challenging clinical scenario.

The findings from this case underscore the rarity of gastric and small bowel metastases originating from lung cancer and highlight the generally poor prognostic outlook associated with such metastatic occurrences. These metastases often manifest aggressive behavior and rapid progression, posing significant challenges in terms of management and treatment. While the long-term benefits of surgical resection for such cases remain uncertain.

In light of the complexities surrounding gastric and small bowel metastases from lung cancer, further research endeavors are warranted to unravel the underlying mechanisms fueling these metastatic events. By advancing our knowledge in this domain, we aim to optimize therapeutic strategies and enhance outcomes for individuals affected by similar metastatic presentations.

## 2. Case description

### 2.1. Patient history

The patient is a 41-year-old male, occupation as a construction worker, with no history of smoking. He sought medical attention on October 21, 2021, for a cough that had lasted for 1 month, without hemoptysis, and with slight chest discomfort.

### 2.2. Imaging examination

**Figure 1.**
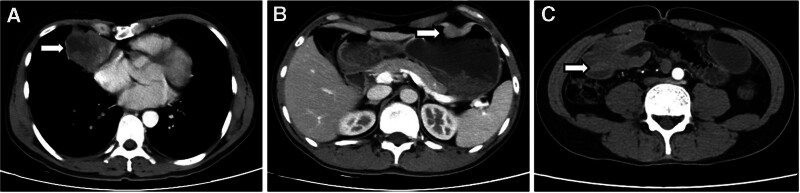
Enhanced CT scan showing a tumor in the right lung (A); malignant tumor on the greater curvature of the stomach (B); intestinal mass and intussusception (C).

Enhanced CT scan of the chest, abdomen, and pelvis revealed a tumor in the right middle lobe of the lung (4.5 cm × 5.6 cm) with metastasis to the right hilar and mediastinal lymph nodes, as well as a mass in the greater curvature of the stomach (3.2 cm × 1 cm) and multiple lymph node metastases in the abdomen (Fig. 1A and B). PET-CT showed the following: (1) peripheral type lung cancer in the right middle lobe with multiple lymph node metastases in the mediastinum and right hilar region, as well as lung metastasis in the left upper lobe and rib metastasis in the 9th rib on the left side. (2) Obstructive inflammation and atelectasis in the right middle lobe of the lung. (3) Malignant tumor in the greater curvature of the stomach with multiple lymph node metastases in the abdomen. Thirty-five days after the initial visit, the patient experienced worsening vomiting symptoms and melena. A follow-up CT scan of the abdomen and pelvis showed gastric body greater curvature side and right middle abdomen intestinal mass (Fig. 1C), resulting in secondary intestinal obstruction and intussusception, as well as multiple enlarged lymph nodes in the abdominal and retroperitoneal regions, suggesting small bowel metastasis based on the patient’s history.

### 2.3. Pathological examination and genetic testing

**Figure 2.**
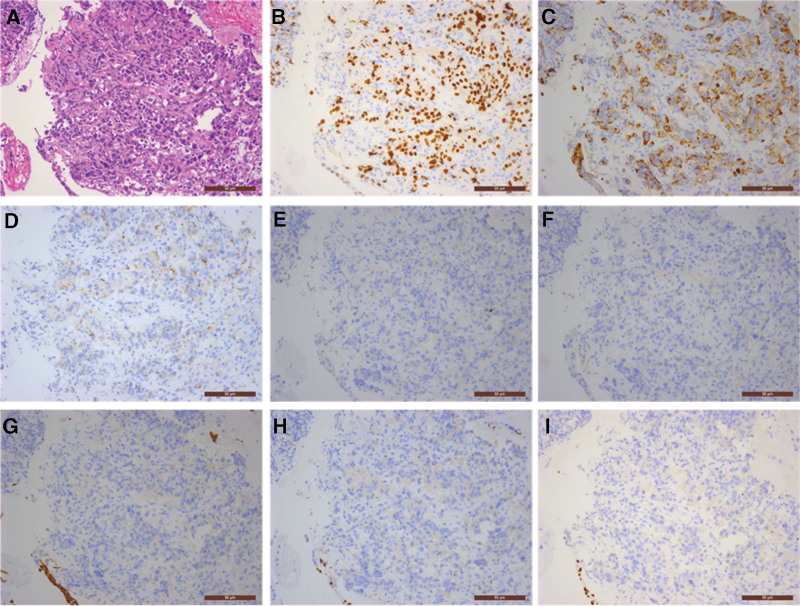
Lung tumor bronchoscopy biopsy pathology (200×): (A) hematoxylin and eosin stain revealed poorly differentiated carcinoma; (B) TTF-1 (+); (C) CK7 (+); (D) Napsin-A (+); (E) CK20 (−); (F) CDX2 (−); (G) CK5/6 (−); (H) p63 (−); (I) P40 (−).

**Figure 3.**
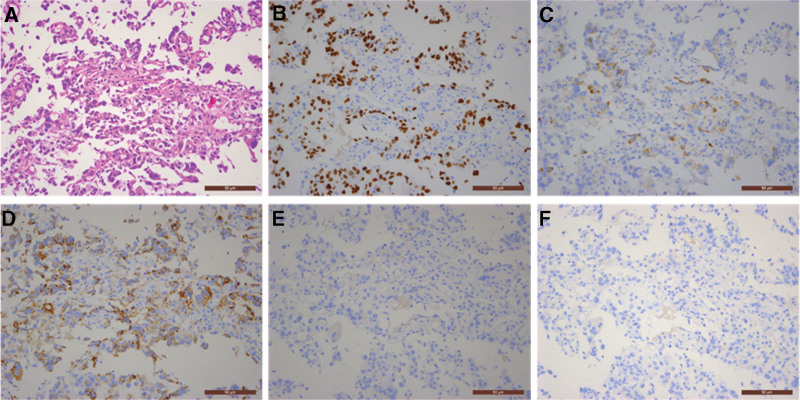
Gastric endoscopy pathology (200×): (A) hematoxylin and eosin stain revealed adenocarcinoma; (B) TTF-1 (+); (C) CK7 (weakly positive); (D) Napsin-A (+); (E) CK20 (−); (F) CDX2 (−).

Bronchoscopy biopsy pathology results showed poorly differentiated adenocarcinoma in the inner side of the right middle lobe, and immunohistochemistry confirmed the diagnosis of poorly differentiated adenocarcinoma; immunohistochemistry staining results were as follows: CK7 (+), CK20 (−), CDX2 (−), TTF-1 (+), Napsin-A (+), CK5/6 (−), p63 (−), P40 (−), and Ki-67 (80%+) (Fig. 2A–I). Genetic testing results showed no mutations or amplification in epidermal growth factor receptor 1, human epidermal growth factor receptor 2, BRAF, anaplastic lymphoma kinase, KRAS, MET, NRAS, RET, TP53, ROS1, MAP2K1, PIK3CA, NTRK1, NTRK2, and NTRK3. Transbronchoscopic needle aspiration biopsy results for lymph nodes in group 7 and 4R showed malignant tumor cells, suggesting non-small cell carcinoma. Gastric endoscopy pathology revealed adenocarcinoma in the gastric body, and based on immunohistochemistry and patient history, the tumor was suspected to originate from lung adenocarcinoma; immunohistochemistry staining results were as follows: TTF-1 (+), Napsin-A (+), CK7 (weakly positive), CDX2 (−), CK20 (−), and Ki-67 (60%+) (Fig. 3A–F).

### 2.4. Treatment and outcome

The family refused surgical intervention. Thirty-five days after the initial consultation, the patient exhibited worsening symptoms of vomiting and melena. A follow-up CT scan revealed small bowel metastasis, leading to secondary intestinal obstruction and intussusception. Additionally, the patient developed persistent hematemesis and severe anemia. Later, the patient received interventional hemostatic treatment; however, the efficacy of the intervention was poor. The symptoms of intestinal obstruction worsened, and the patient eventually died 78 days after the initial visit.

## 3. Discussion

The gastric and small bowel metastases are rare, respectively 0.4% and 1.1% and the cases reported in the literature are rare.^[1]^ The occurrence of lung cancer spreading to gastrointestinal sites in a clinical setting is reported to be as minimal as 0.2% to 1.7%.^[2–4]^ Gastric and small bowel metastases from lung cancer are more common in middle-aged and elderly male smokers; the clinical symptoms usually include abdominal pain, melena, obstruction, and vomiting; CT commonly shows gastric and small bowel masses, with submucosal tumors being more common in the stomach, ulcers may also be present.^[5,6]^ The mechanism of lung cancer metastasizing to the gastrointestinal tract is currently unclear. It is theorized that lung cancer can lead to gastric metastasis through the ingestion of sputum containing cancer cells that then migrate to the digestive tract. This process is particularly significant in smokers, as they are more prone to gastric mucosal injury compared to nonsmokers.^[7]^ Owing to the scarcity of cases, the long-term benefit of surgical resection is uncertain. However, for recurrent and difficult-to-manage bleeding or obstruction, proactive surgical treatment is still recommended.^[2,8]^ In addition, aggressive surgical intervention for a solitary gastric metastatic lesion has shown survival benefits,^[9,10]^ and is necessary for preventing major bleeding and perforation.^[9,11]^ However, patient selection is crucial, taking into consideration the patient’s general condition, whether it is a solitary lesion, and whether surgery is needed to control complications. The surgical approach should be determined based on the clinical situation. Research has shown that in the case of solid tumor patients with gastrointestinal metastasis, the median survival after the diagnosis of gastric metastasis was 8.53 months.^[6]^ Another study on gastrointestinal metastasis of primary lung cancer revealed that the mean time between identifying gastrointestinal metastasis and mortality was 100.6 days (range, 21–145 days).^[4]^ The presence of gastrointestinal metastasis is likely to be a preterminal event, indicating a poor prognosis and an extremely short survival period.^[12]^ Overall, the prognosis for gastric and small bowel metastasis from lung cancer is poor.

In this case, the patient was found to have both lung tumors and gastric masses during the examination, immunohistochemistry examination was primarily used to clarify the tumor’s origin and type. The results of the lung tumor showed positive for TTF-1, Napsin-A, and CK7, while negative for CDX2 and CK20, ruling out the possibility of gastrointestinal origin and indicating primary lung cancer. Furthermore, the negative results for CK5/6, p63, and P40 excluded squamous cell carcinoma, and in combination with the tumor tissue morphology observed in the H&E slides, the pathological diagnosis was determined to be poorly-differentiated adenocarcinoma. It is worth noting that Ki-67 showed 80% positivity, suggesting active proliferation of tumor cells with a high malignant grade. The immunohistochemistry results for the gastric tumor were consistent with the lung tumor, also showing positivity for TTF-1, Napsin-A, and CK7, and negativity for CDX2 and CK20. Therefore, considering the patient’s medical history, the primary consideration was lung adenocarcinoma origin. Additionally, Ki-67 was 60% positive, indicating a high proliferation activity similar to the primary lesion. The progression of the patient’s condition confirmed the highly malignant nature of the tumor and its rapid advancement. At 35 days after the initial visit, a follow-up CT examination revealed small bowel metastasis with obstruction. Due to the patient experiencing persistent hematemesis and severe anemia, and refusing surgical treatment, the patient only received interventional hemostasis treatment to alleviate symptoms but with poor efficacy. Ultimately, the patient passed away 78 days after the initial visit.

## 4. Conclusion

We present a case of a male patient with lung adenocarcinoma metastasizing to the stomach and subsequently developing small bowel metastasis during treatment. The patient’s bronchoscopy biopsy, combined with immunohistochemistry results, confirmed primary lung adenocarcinoma. Gastric endoscopy pathology indicated that the gastric mass originated from lung adenocarcinoma. The patient’s condition progressed rapidly, and at 35 days after the initial hospitalization, small bowel metastasis with obstruction was identified. Whether surgical resection, if performed, could impact the disease trajectory of this patient remains unknown. Further research is needed to enhance our understanding of the mechanisms behind these metastatic events and improve treatment options for affected individuals.

## Author contributions

**Conceptualization:** Yan Wang.

**Data curation:** Yan Wang, Li Xu, Jun-Lei Wu.

**Writing – original draft:** Yan Wang.

**Writing – review & editing:** Chao-Qun Wang.
